# Antimicrobial susceptibility of bacterial isolates from ambulatory practice and from a referral hospital

**DOI:** 10.1111/jvim.15685

**Published:** 2019-12-17

**Authors:** Julie F. N. Potier, Andy E. Durham

**Affiliations:** ^1^ The Liphook Equine Hospital Hampshire United Kingdom

**Keywords:** antirobial, horse, MIC, practice, resistance

## Abstract

**Background:**

Responsible use of antimicrobials in equine practice relies on knowledge of common bacterial isolates and their antimicrobial sensitivities.

**Objectives:**

To assess the frequency of bacterial resistance to a combination of parenteral penicillin and gentamicin and to trimethoprim and sulfamethoxazole for PO use in a selection of clinical isolates, and subsequently to determine the prevalence of resistance to antimicrobials that might then be used as alternatives to first‐line antimicrobials for the same isolates.

**Methods:**

Retrospective analysis of minimal inhibitory concentrations (MICs) of antimicrobials for 6354 bacterial isolates from 365 ambulatory practices and 519 isolates from a referral hospital. The MICs were used to indicate sensitivity or resistance to commonly used antimicrobials and the prevalences of resistance were compared between origin of the isolates, and among antimicrobial drugs.

**Results:**

Isolates from the referral hospital were significantly (*P* < .05) more likely to be resistant to the antimicrobials tested than those derived from ambulatory practice. Overall, 91% of the ambulatory isolates and 64% of the hospital isolates were sensitive to penicillin‐gentamicin. For trimethoprim‐sulfamethoxazole combination, 82% of the ambulatory practice isolates and 56% of the referral hospital isolates were sensitive.

**Conclusions and Clinical Importance:**

Most isolates were sensitive to penicillin and gentamicin as well as trimethoprim‐sulfamethoxazole. No predictable efficacious second choice antimicrobial was identified for those isolates resistant to the first‐line antimicrobials. The likelihood of isolates being sensitive to second choice antimicrobials was variable but generally higher for ambulatory isolates compared to referral isolates. Bacterial identification and measurement of MIC are essential to make the appropriate antimicrobial choice.

**Abbreviations**
CLSI: Clinical and Laboratory Standards Institute
EUCAST: European Union Committee on Antimicrobial Susceptibility Testing
MIC: minimal inhibitory concentration
P‐G: procaine penicillin with gentamicin sulfate combination
TMPS: trimethoprim‐sulfamethoxazole

## 1 INTRODUCTION

Antimicrobial use in veterinary practice has been linked to increased resistance in bacterial infections in both human and veterinary healthcare,^1^ and most especially in hospital practice.^2^, ^3^ Clinicians should apply principles of antimicrobial stewardship when considering their choice of antimicrobials,^4^ which is not necessarily always the case currently in the United Kingdom (UK).^5^ In some other European countries such as Sweden or Denmark, critically important antimicrobials have legally enforced restricted use in veterinary medicine.^6^

The British Equine Veterinary Association guidelines for responsible antimicrobial use advise that first‐line injectable antimicrobials should be a combination of procaine penicillin with gentamicin sulfate (P‐G) in most infectious scenarios encountered in equine practice (http://www.beva.org.uk/protectme). Among the PO antimicrobials available, a trimethoprim‐sulfadiazine (TMPS) combination is recommended for first‐line use^7^ and is also the only licensed choice in horses in the UK.

Responsible use of antimicrobials is facilitated by in vitro antimicrobial susceptibility testing to inform antimicrobial choice. Breakpoints are determined based on the minimum inhibitory concentration (MIC) of an antimicrobial to separate isolates for which there is a high likelihood of treatment success (sensitive) versus those for which treatment is more likely to be ineffective (intermediate or resistant).^8^, ^9^ International groups including the Clinical and Laboratory Standards Institute (CLSI) and the European Union Committee on Antimicrobial Susceptibility Testing (EUCAST) have published suggested breakpoints for clinical application based on pharmacokinetics and pharmacodynamics to predict clinical efficacy.^10^, ^11^ However, very little specific data are available for horses, requiring some extrapolation from other species.^12^

Our aim was to examine the value of in vitro antimicrobial sensitivity testing in antimicrobial selection in both ambulatory and hospital practice. The prevalence of sensitivity to first‐line antimicrobials (parenteral P‐G and PO TMPS) was determined and subsequently the sensitivity to alternative antimicrobials was examined for the isolates found to be resistant to first‐line choices. The hypotheses were that bacteria cultured from a hospital population would have lower prevalence of in vitro sensitivity compared to ambulatory isolates as previously described^9^ and that organisms resistant to P‐G or TMPS still could be treated successfully with noncritically important antimicrobials such as tetracyclines.

## 2 MATERIALS AND METHODS

In this retrospective analysis, data were collected from all clinical samples submitted to The Liphook Equine Hospital Laboratory for culture and determination of antimicrobial sensitivity between January 2014 and December 2018.

Submitted samples were plated onto Columbia blood agar and MacConkey's agar, colistin‐nalidixic acid agar, or combinations thereof depending on sample type. Subcultures usually were prepared, depending on the purity of primary growth, to obtain pure cultures before suspending individual colonies in saline to a McFarlane standard of 0.5. The suspension then was processed using a VITEK 2 analyzer (BioMerieux, Basingstoke, Hampshire, UK) for bacterial identification. Isolates were further examined after separating Gram‐negative and Gram‐positive bacteria using a different array of antimicrobials to determine MICs (AST‐GN65, AST‐GP73, BioMerieux). Antimicrobial susceptibilities were examined across a range of dilutions from 0.5 to 16 μg/mL for gentamicin, 0.06 to 16 μg/mL for benzylpenicillin, 0.1 to 320 μg/mL for trimethoprim‐sulfamethoxazole (because sulfadiazine was not available for susceptibility testing on the analyzer used), 0.25 to 16 μg/mL for tetracycline, 0.25 to 8 μg/mL for ceftiofur, and 0.25 to 4 μg/mL for enrofloxacin. Antimicrobial breakpoints were selected from data provided by EUCAST and CLSI and used to categorize isolates as sensitive or resistant. For the purposes of analysis, isolates with MICs categorized as intermediate susceptibility were classified as resistant.^11^

All of the samples were categorized into different main anatomic sites of origin: skin/wounds (including fistulous withers, surgical incisions and IV catheters), respiratory (pleural fluid, bronchoalveolar lavage, tracheal wash, guttural pouch lavage, sinus fluid, nasal discharge), reproductive female (cervix, clitoris, fetus, uterus, vulvar or vaginal discharge), abscesses (including dental, foot or septic pedal bone), urinary (bladder biopsy, urine), ocular, other (surgical implants; internal biopsy samples such as intestines, liver, stomach, ovary, peritoneal fluid, synovial fluid, penile swab, mammary discharge, diarrhea), or unknown origin.

The population of isolates found to be resistant to P‐G was evaluated to determine the prevalence of sensitivity to other antimicrobials that might be selected as a second choice in practice consisting of TMPS, tetracycline, ceftiofur, and enrofloxacin. Not all isolates were tested against every antimicrobial, given that some would not be logical choices based on known pharmacodynamics. For example, gram‐negative bacteria were not tested for sensitivity to penicillin. Except for *Enterobacter spp*, *Enterococcus spp* was not tested for ceftiofur, and *Streptococcus spp* was not tested for enrofloxacin and gentamicin.^13^, ^14^

The population of isolates found to be resistant to TMPS was evaluated to determine the prevalence of sensitivity to other antimicrobials that might be selected as a second choice in practice based on known pharmacodynamics comprising tetracycline, ceftiofur, enrofloxacin, or P‐G.

### 2.1 Statistical analysis

Bacterial resistance to antimicrobials was compared between samples derived from ambulatory practices and those obtained from the referral hospital by using a Chi‐squared test when appropriate (>5 expected cases) or a Fisher's exact test, with a *P* value <.05 indicating a significant difference.

The prevalences of resistance to the second‐choice antimicrobials in ambulatory and hospital practice also were compared with one another using a Chi‐squared test when appropriate (>5 isolates) or a Fisher's exact test, with a *P* value <.05 indicating a significant difference.

GraphPad Prism 8 software (GraphPad Prism version 8.0.0 for Windows, GraphPad Software, San Diego, California, http://www.graphpad.com) was used, with contingency tables to compare the data in ambulatory practice and in the referral hospital population, as well as second‐choice antimicrobials in pairs.

## 3 RESULTS

A total of 6873 isolates were identified and their MICs determined during the study period. Of these, 6354 (92%) came from 345 different ambulatory practices and 519 (8%) came from a single referral hospital population.

### 3.1 Comparison of ambulatory and referral isolates

Overall 5685 (91%) of the 6354 ambulatory isolates were sensitive to P‐G (Table 1), and 4833 (82%) were sensitive to TMPS (Table 2). Of the 519 hospital isolates, 314 (64%) were sensitive to P‐G (Table 3), and 275 (56%) were sensitive to TMPS (Table 4).

**Table 1:** Six thousand two‐hundred fifty isolates from 345 ambulatory practices and 491 isolates from a referral hospital tested for Penicillin‐Gentamicin (P‐G) sensitivity alongside prevalence of sensitivity (F, female)

Penicillin‐Gentamicin	Total ambulatory	P‐G tested	P‐G sensitive		Total referral	P‐G tested	P‐G sensitive	
			n	%			n	%
Skin/wounds	2151	2137	1915	89	291	271	135	50
Respiratory	1267	1251	1148	92	108	106	88	83
Reproductive F	330	305	280	92	5	5	5	100
Abscesses	790	764	708	93	54	52	45	87
Urinary	242	220	207	94	11	8	4	50
Ocular	170	168	155	92	4	4	4	100
Other	324	319	300	94	34	33	24	73
Unknown	1094	1072	972	91	12	12	9	75
Total	6354	6250	5585	91	519	491	314	64

**Table 2:** Five thousand nine‐hundred thirty isolates from 345 ambulatory practices and 489 isolates from a referral hospital tested for Trimethoprim‐Sulfamethoxazole (TMPS) sensitivity alongside prevalence of sensitivity (F, female)

Trimethoprim‐sulfamethoxazole	Total ambulatory	TMPS tested	TMPS sensitive		Total referral	TMPS tested	TMPS sensitive	
			n	%			n	%
Skin/wounds	2137	2001	1490	74	291	278	134	48
Respiratory	1267	1192	1035	87	108	99	74	75
Reproductive F	330	314	248	79	5	4	0	0
Abscesses	790	735	628	85	54	48	32	87
Urinary	242	216	167	77	11	11	0	0
Ocular	170	145	137	94	4	4	4	100
Other	324	287	244	85	34	34	23	68
Unknown	1094	1040	884	85	12	11	8	73
Total	6354	5930	4833	82	519	489	275	56

**Table 3:** Six‐hundred sixty five ambulatory isolates found to be resistant to Penicillin‐Gentamicin (P‐G) and tested for sensitivity to alternative antimicrobials (F, female; TMPS, trimethoprim sulfamethoxazole combination)

Ambulatory practice P‐G resistant	TMPS tested	TMPS sensitive		Tetra‐cycline tested	Tetracycline sensitive		Ceftio‐fur tested	Ceftiofur sensitive		Enro‐floxacin tested	Enrofloxacin sensitive	
		n	%		n	%		n	%		n	%
Skin/wounds	230	59	26	234	39	17	134	61	46	220	118	54
Respiratory	94	73	78	101	63	62	35	18	51	40	24	60
Reproductive F	22	11	50	25	7	28	16	6	38	18	14	78
Abscesses	51	25	49	56	22	39	29	16	55	44	26	59
Urinary	9	6	66	13	6	46	5	1	20	8	2	25
Ocular	10	5	50	12	6	50	3	2	67	8	4	50
Other	18	8	44	16	6	38	10	6	60	13	6	46
Unknown	95	51	54	100	47	47	37	19	51	52	34	65
Total	529	236	46	557	196	35	269	129	48	403	228	57

**Table 4:** One thousand ninety seven ambulatory isolates found to be resistant to Trimethoprim‐sulfamethoxazole (TMPS) and tested for sensitivity to alternative antimicrobials (F, female; P‐G, Penicillin‐Gentamicin association)

Ambulatory practice TMPS resistant	Tetra‐cycline tested	Tetracycline sensitive		Enro‐floxacin tested	Enrofloxacin sensitive		Ceftio‐fur tested	Ceftiofur sensitive		P‐G tested	P‐G sensitive	
		n	%		n	%		n	%		n	%
Skin/wounds	515	133	26	496	301	61	366	227	62	506	301	60
Respiratory	150	90	60	68	40	59	114	90	79	158	125	79
Reproductive F	68	34	50	58	47	81	49	29	73	64	51	80
Abscesses	110	35	32	92	71	77	68	45	66	104	77	74
Urinary	50	33	66	48	30	63	24	20	83	43	40	93
Ocular	9	5	56	9	6	67	6	5	83	9	5	56
Other	44	17	39	40	29	73	31	20	65	41	30	73
Unknown	157	63	40	108	75	69	87	35	40	156	110	71
Total	1103	410	37	919	599	65	745	451	61	1081	739	68

Prevalence of resistance to P‐G was significantly different between ambulatory and referral isolates for all isolates combined (*P* < .001) as well as for the subcategories of skin/wounds (*P* < .001), respiratory (*P* < .001), reproductive female (*P* < .001), abscesses (*P* < .001), ocular (*P* < .001), other (*P* = .05), and unknown (*P* < .001).

Prevalence of resistance to TMPS was significantly different between ambulatory and referral isolates for all isolates combined (*P* < .001), as well as for the subcategories of skin/wounds (*P* = .003), respiratory (*P* < .001), reproductive female (*P* = .01), urinary (*P* < .001), ocular (*P* < .001), other (*P* < .001), and unknown (*P* < .001).

### 3.2 Comparison of antimicrobials selected as second choice to resistant isolates

The prevalence of sensitivity to the various second‐choice antimicrobials isolates found to be resistant to P‐G or TMPS are listed in Tables 3, 4, 5, 6. A significant difference (*P* < .05) was found between the prevalence of resistance to each antimicrobial used when compared in pairs for all except TMPS and ceftiofur (*P* = .41), enrofloxacin and P‐G (*P* = .14), and ceftiofur and enrofloxacin (*P* = .59) for the ambulatory samples. A significant difference also was found between the rates of resistance to each antimicrobial used when compared in pairs for all except TMPS and tetracycline (*P* = .46), and ceftiofur and enrofloxacin (*P* = .38) for the hospital isolates.

**Table 5:** One‐hundred seventy seven referral hospital isolates found to be resistant to Penicillin‐Gentamicin (P‐G) and tested for sensitivity to alternative antimicrobials (F, female; TMPS, trimethoprim sulfamethoxazole combination)

Referral hospital P‐G resistant	TMPS tested	TMPS sensitive		Tetra‐cycline tested	Tetracycline sensitive		Ceftio‐fur tested	Ceftiofur sensitive		Enro‐floxacin tested	Enrofloxacin sensitive	
		n	%		n	%		n	%		n	%
Skin/wounds	130	14	11	136	11	8	96	31	32	136	46	34
Respiratory	17	2	22	18	2	11	9	4	44	16	10	63
Reproductive F	0	0	0	0	0	0	0	0	0	0	0	0
Abscesses	6	2	33	6	1	17	3	0	0	7	3	43
Urinary	4	0	0	4	0	0	4	0	0	4	3	75
Ocular	0	0	0	0	0	0	0	0	0	0	0	0
Other	10	0	0	10	0	0	7	0	0	9	6	67
Unknown	2	0	0	3	0	0	1	0	0	3	2	67
Total	169	18	11	177	14	8	120	35	29	175	70	40

## 4 DISCUSSION

We found that bacterial isolates collected from ambulatory practice were more likely to be sensitive to P‐G and to TMPS than those collected from a referral hospital. We also found that where resistance to first‐line antimicrobials was found, no second‐choice antimicrobial was consistently predicted to be efficacious, with <68% of the isolates resistant to P‐G or TMPS being found to be sensitive to any other antimicrobial.

The finding of higher resistance rates in isolates from a referral hospital compared to those obtained from ambulatory practices (Tables 1, 2, 3, 4) also has been found in previous studies.^9^, ^15^ This observation probably can be explained because of higher antimicrobial exposure among a hospital bacterial population because resistance genes are put under more environmental pressure as well as other factors such as greater potential for transmission of resistant strains or resistance determinants among hospitalized horses and for stress to precipitate increased shedding of resistant strains.^2^ It is especially important to establish MICs for isolates from within a hospital population because of a generally lower likelihood of antimicrobial efficacy. By the same reasoning, it is logical that we found protected antimicrobials such as enrofloxacin and ceftiofur to have a lower prevalence of resistance among isolates because they are used less commonly (Tables 5‐8). This latter finding might be used incorrectly as justification to employ these antimicrobials more frequently as first‐line choices for bacterial infections although the inevitable consequence of such action would be to rapidly increase resistance prevalence to these antimicrobials, leaving little choice thereafter.

The array of second‐line antimicrobials was selected based on common equine clinical practice and also availability of these drugs in the UK. Enrofloxacin and doxycycline however are not licensed for use in horses in the UK, and ceftiofur is only licensed for bacterial respiratory diseases associated with *Streptococcus spp*, *Staphylococcus spp*, and *Pasteurella spp*. Therefore, when second‐line antimicrobials are to be selected, preference should be given licensed choices such as oxytetracycline, P‐G, and TMPS, which have been shown to have adequately low MICs. Ceftiofur might be a reasonable choice in the case of isolates found to be resistant to oxytetracycline, P‐G, and TMPS, especially in the case of respiratory disease. Doxycycline might be reasonably selected as a second‐line antimicrobial when P‐G and TMPS resistance is found, when for reasons of practicality or safety parenteral antimicrobial administration is considered unsuitable or both. Enrofloxacin should be conserved on both legal and medical grounds and should only be used when none of the aforementioned antimicrobials is considered suitable based on MIC data. Enrofloxacin has a relatively limited expected spectrum of activity and is not a reasonable choice for most anerobic or streptococcal infections.^13^, ^14^

In ambulatory cases where MIC data predicted likely failure of P‐G treatment, the predicted success of second‐choice antimicrobials varied from 35% for tetracyclines to 57% for enrofloxacin, whereas for hospital isolates the equivalent figures were 8% for tetracyclines to 40% for enrofloxacin (Tables 5 and 7). In ambulatory cases where MIC data predicted likely failure of TMPS treatment, the predicted success of second choice antimicrobials varied from 37% for tetracycline to 68% for P‐G, whereas for hospital isolates the equivalent figures were 23% for tetracyclines to 55% for enrofloxacin (Tables 6 and 8). In ambulatory practice, there may be greater indication for PO antimicrobials for reasons of practicality and therefore preferences for TMPS, doxycycline, or enrofloxacin. Although enrofloxacin showed lower rates of resistance among the isolates from ambulatory cases compared to the other PO drugs, the rates of resistance nevertheless were high enough to prevent any confidence that enrofloxacin would be efficacious in the absence of determining MIC data. Also, enrofloxacin is not licensed for use in horses, is firmly within the group of critically important antimicrobials, and therefore should be used only with very good evidence‐based reasoning. For hospital referral practice, parenteral administration is rarely problematic, meaning that parenteral P‐G frequently is used as a first‐line choice, and parenteral TMPS or oxytetracycline could be suitable second‐choice antimicrobials where resistance is seen to P‐G. Although sensitivity rates of P‐G‐resistant isolates generally were quite poor to TMPS and tetracyclines (11 and 8%, respectively, Table 7), these drugs nonetheless should be selected when the MIC is found to be below the clinical breakpoints or when other circumstances exist that might promote efficacy of these drugs (eg, local application). Although where resistance is seen to P‐G, TMPS, and oxytetracycline, the further choices of ceftiofur or enrofloxacin might be considered, the sensitivity rates of isolates to these 2 further protected antimicrobials were only 29 and 40%, respectively, reinforcing the fact that they would be poor speculative choices and only should be used based on MIC data.

**Table 6:** Two‐hundred fourteen referral hospital isolates found to be resistant to Trimethoprim‐sulfamethoxazole (TMPS) and tested for sensitivity to alternative antimicrobials (F, female; P‐G, Penicillin‐Gentamicin association)

Referral hospital TMPS resistant	Tetra‐cycline tested	Tetracycline sensitive		Enro‐floxacin tested	Enrofloxacin sensitive		Ceftio‐fur tested	Ceftiofur sensitive		P‐G tested	P‐G sensitive	
		n	%		n	%		n	%		n	%
Skin/wounds	143	28	20	139	69	50	84	38	45	141	41	29
Respiratory	24	5	21	23	14	61	17	9	53	25	13	52
Reproductive F	0	0	0	0	0	0	0	0	0	0	0	0
Abscesses	16	8	50	15	10	67	12	7	58	15	9	60
Urinary	12	6	50	12	6	50	10	2	20	10	2	20
Ocular	0	0	0	0	0	0	0	0	0	0	0	0
Other	11	2	18	11	9	82	16	12	75	11	1	9
Unknown	3	0	0	2	1	50	1	1	100	3	1	33
Total	209	49	23	202	110	55	140	69	49	205	67	33

Strict application of MIC data to predict clinical efficacy or inefficacy sometimes may mislead because the many assumptions underlying the prediction of sensitivity and resistance might not always be correct. The so‐called 60‐90 rule often is quoted as a guide, which states that bacterial infections with in vitro prediction of efficacy to a particular antimicrobial will resolve in 90% of patients treated with that antimicrobial, whereas 60% of bacterial isolates with predicted resistance still might respond well.^16^ There are many instances and reasons why bacteria with apparent in vitro resistance to an antimicrobial actually might respond well clinically to that antimicrobial. These factors include the host's own immunity, which contributes to bacterial clearance, but also pharmacokinetic properties that might favor antimicrobial accumulation at a particular site of infection. For example, urinary excretion of TMPS and P‐G will lead to especially high urinary concentrations and clinical efficacy against urinary tract isolates even when in vitro testing might suggest resistance.^17^, ^18^ This divergence between in vitro test results and clinical efficacy is likely to be even more pronounced for infections that are amenable to topical or local treatments (eg, intra‐ocular, intra‐uterine, dermal, IV regional perfusion) because local concentrations will be many times higher than could be achieved by systemic administration. For example, an isolate with an MIC for gentamicin of 16 μg/mL would be predicted to be highly resistant based on efficacy requiring attainment of 128 to 160 μg/mL gentamicin (8‐10 times the MIC) in the locality of the infection, which is unachievable with systemic administration.^9^ However, such local concentrations are relatively easily achievable by topical treatment.^19^ Local treatments also will decrease the incidence of adverse effects on the fecal microbiota and the development of antimicrobial‐associated diarrhea.^20^ Another important consideration is the synergistic action of some antimicrobial combinations leading to better clinical efficacy than would be expected based on the spectrum of each antimicrobial taken separately.^21^ For example, trimethoprim and penicillin demonstrate synergism when given along with aminoglycosides.^22^

Conversely, there are instances and reasons why bacteria with apparent in vitro sensitivity to an antimicrobial may not respond well clinically to that antimicrobial. Infections may occur at sites poorly accessible to the chosen antimicrobial or potentially antagonistic factors may impair the pharmacodynamic properties of the antimicrobial in question.^23^, ^24^, ^25^ Additionally, suppressive or antagonistic drug interactions can occur when poorly selected polypharmacy is employed.^26^ A further intriguing strategy for improving antimicrobial sensitivity involves the concept of collateral drug sensitivity where a bacterial strain that has acquired resistance to 1 class of antimicrobials (especially aminoglycosides) may sometimes simultaneously become more sensitive to other antimicrobials.^27^, ^28^, ^29^

Prudent use of antimicrobials hopefully will promote good efficacy in clearing bacterial infections while limiting the increase in bacterial resistance.^30^ Many facets contribute to achieving this balance beginning with correct identification of the specific pathogenic threats, and followed by careful selection of appropriate antimicrobials, which requires awareness of their pharmacokinetic and pharmacodynamic properties as well as their MICs for specific pathogens. Prioritization of nonprotected antimicrobials always should be practiced where supported by the considerations discussed above, thus conserving additional antimicrobials for clinical situations for which no other choices exist. Selection of antimicrobials in the absence of supportive data increases the risk of inefficacy along with subtherapeutic exposure, which contributes to the prevalence of resistance. When budgetary constraints dictate speculative selection, there is rarely any justification for the use of protected antimicrobials.

Our study reconfirmed previous evidence of higher rates of antimicrobial resistance in isolates from an equine hospital compared to ambulatory practices. Additionally, we found that when resistance was found to first‐line antimicrobial choices, the choice of subsequent antimicrobials could not be predicted with confidence and always should be based on MIC data rather than speculation.

## CONFLICT OF INTEREST DECLARATION

Authors declare no conflict of interest.

## OFF‐LABEL ANTIMICROBIAL DECLARATION

Authors declare no off‐label use of antimicrobials.

## INSTITUTIONAL ANIMAL CARE AND USE COMMITTEE (IACUC) OR OTHER APPROVAL DECLARATION

This was a retrospective study of clinical data for which prior consent was obtained.

## HUMAN ETHICS APPROVAL DECLARATION

Authors declare human ethics approval was not needed for this study.
